# Genetic Burden and *APOE* Methylation in a Korean Multi-Generational Alzheimer’s Disease Family: An Exploratory Multi-Omics Case Study

**DOI:** 10.3390/jpm16020066

**Published:** 2026-01-29

**Authors:** Je-Hyun Eom, Mu-Yeol Cho, Ji-Won Kim, Yunwoo Kim, Seung-Jo Yang, Jiyoung Hwang, Dahye Lee, Hye-Sung Kim, Young-Youn Kim, Hanseung Baek

**Affiliations:** 1 Apple Tree Institute of Biomedical Science, Apple Tree Medical Foundation, Goyang-si 10447, Republic of Korea; djawpgus@appleden.com (J.-H.E.); whanduf@appleden.com (M.-Y.C.); rlawldnjs@appleden.com (J.-W.K.); rladbsdn@appleden.com (Y.K.); drdahaelee@appleden.com (D.L.); hyesungk2008@appleden.com (H.-S.K.); trynow@gmail.com (Y.-Y.K.); 2 DOCSMEDI Co., Ltd., Goyang-si 10447, Republic of Korea; didtmdwh@appleden.com (S.-J.Y.); ghkdwldud@appleden.com (J.H.)

**Keywords:** precision medicine, personalized medicine, Alzheimer’s disease, genetic burden score, DNA methylation, *APOE*, multi-omics, risk stratification, family study, Korean population

## Abstract

**Background/Objectives**: Alzheimer’s disease (AD) exhibits high heritability (60–80%), yet individual-level genetic risk prediction remains challenging. While *APOE* ε4 is the strongest genetic risk factor, incomplete penetrance complicates risk assessment. **Methods**: We analyzed seven blood-related members across three generations using the Korean Chip v2.0 genotyping (~1.2 M SNPs) and Illumina EPICv2 DNA methylation profiling. Genetic burden score (GBS) was calculated by summing risk alleles across 320 variants in six AD-associated genes (*APOE*, *PICALM*, *CLU*, *CR1*, *BIN1*, and *ABCA7*). **Results**: An unexpected pattern was observed in this family: the affected individual (J-003) had the lowest GBS (39 alleles), while individuals with higher genetic burden (51–61 alleles) remained cognitively healthy. J-003 also exhibited lower *APOE* methylation (β = 0.495) compared to the family mean (β = 0.523). *CR1* contributed the most risk alleles across the family, followed by *PICALM*. **Conclusions**: This single-case observation cannot establish causality, generalizability, or biological significance. The affected individual’s lower APOE methylation may represent a causal factor, disease consequence, or coincidental variation—scenarios that cannot be distinguished from this dataset. Validation in larger cohorts with multiple affected individuals is required to determine whether integrated multi-omics approaches can inform personalized risk assessment in familial contexts.

## 1. Introduction

Alzheimer’s disease (AD) is the most prevalent form of dementia, affecting over 55 million people worldwide, with projections indicating this number will triple by 2050 [1]. The disease’s high heritability (60–80%) [2,3] makes understanding its genetic architecture essential for developing predictive biomarkers and targeted therapeutic interventions—a core principle of precision medicine. The *APOE* gene, particularly the ε4 allele, represents the strongest genetic risk factor for late-onset AD. Compared to ε3/ε3 carriers, individuals with one ε4 allele face a 3-fold increased risk, while homozygous ε4/ε4 carriers exhibit up to a 15-fold elevated risk [4]. However, incomplete penetrance—with many carriers remaining cognitively intact while some non-carriers develop dementia—highlights the complexity of AD genetics. GWAS have identified additional susceptibility loci, including *PICALM*, *CLU*, *CR1*, *BIN1*, and *ABCA7* [5], enabling polygenic risk score development for population-level risk stratification [6]. However, a critical gap remains between population-level associations and individual-level prediction accuracy: why do some individuals with high genetic risk remain cognitively intact, while others with low genetic burden develop dementia [7]? Emerging evidence suggests that epigenetic modifications, particularly DNA methylation, may bridge this gap by dynamically regulating gene expression in response to genetic background and environmental exposures [8]. Notably, altered *APOE* methylation patterns have been associated with AD risk and pathology, potentially explaining why genetic risk scores alone may not fully capture individual disease susceptibility [9,10].

Family-based studies offer unique advantages for dissecting the interplay between genetic and epigenetic factors in complex diseases [11]. Unlike population-based cohorts, family studies control for shared environmental and genetic backgrounds, enabling clearer identification of disease-causing variants and their transmission patterns across generations. Furthermore, the observation of discordant phenotypes within families—where siblings or relatives with similar genetic backgrounds exhibit markedly different disease outcomes—provides natural experiments to identify protective or risk-modifying factors [12].

Despite the growing body of research on AD genetics, studies integrating both genetic and epigenetic data within familial contexts remain scarce, particularly in non-European populations. East Asian populations, including Koreans, exhibit distinct genetic architectures and allele frequency distributions compared to European populations [13], yet comprehensive multi-generational Korean family studies combining genotyping and methylation profiling are lacking. The present exploratory study addresses this gap by investigating a three-generation Korean family cohort comprising seven blood-related members, including one individual with dementia. We performed comprehensive genomic and epigenomic profiling using the Korean Chip v2.0 platform and Illumina EPICv2 DNA methylation analysis. We aim to perform the following: (1) characterize genetic burden across six established AD-associated genes; (2) examine *APOE* methylation patterns; and (3) describe risk allele segregation across generations. This integrated multi-omics approach generates hypotheses about whether epigenetic patterns relate to phenotypic variability beyond genetic burden scores in familial contexts [14,15].

## 2. Materials and Methods

### 2.1. Study Design and Participants

This pilot study investigated a three-generation Korean family cohort comprising seven blood-related members (three grandparents, two parents, and two offspring). One grandparent (J-003, *APOE* ε3/ε4) had been clinically diagnosed with Alzheimer’s disease (AD), while all other family members remained cognitively normal at the time of sample collection. All participants provided written informed consent prior to enrollment. The study was conducted in accordance with the Declaration of Helsinki and approved by the Institutional Review Board of Apple Tree Dental Hospital (protocol code ATDH-2024-0005, approved on 26 September 2024).

Note: The broader family pedigree included 25 members across three generations. However, the present analysis focuses on seven blood-related members with complete genomic and epigenomic data. Spouses were excluded from genetic analyses as they do not share a familial genetic background.

Comprehensive clinical phenotyping was performed for all participants, including cognitive status assessment, blood biochemistry (fasting glucose, lipid profiles including total cholesterol, LDL-cholesterol, HDL-cholesterol, and triglycerides), cardiovascular measurements (blood pressure, body mass index), periodontal health evaluation, and detailed family medical history documentation.

### 2.2. Sample Collection and DNA Extraction

Peripheral blood samples (10 mL) were collected from all seven participants via venipuncture into EDTA-containing tubes. Genomic DNA was extracted from whole blood using the QIAamp DNA Blood Maxi Kit (Qiagen, Hilden, Germany) according to the manufacturer’s protocol. DNA concentration was measured using a NanoDrop 2000 spectrophotometer (Thermo Fisher Scientific, Waltham, MA, USA), and DNA integrity was assessed by agarose gel electrophoresis. DNA samples with A260/A280 ratios between 1.8 and 2.0 and concentrations ≥ 50 ng/μL were used for downstream analyses.

### 2.3. Genotyping Using Korean Chip v2.0

#### 2.3.1. Korean Chip v2.0 Platform

Genome-wide genotyping was performed using the Korean Chip v2.0 (Korea Disease Control and Prevention Agency, Cheongju, Republic of Korea), a next-generation SNP array optimized for Korean population genetics and precision medicine research. The Korean Chip v2.0 was specifically designed to capture Korean-specific low-frequency and rare variants (minor allele frequency <1%) that are underrepresented in European-centric platforms, with enhanced coverage for functional variants relevant to clinical diagnosis and drug repurposing studies.

#### 2.3.2. Genotyping Procedure

Genotyping services were provided by JSLink, Inc. (31 Magokjungang 8-ro 3-gil, Gangseo-gu, Seoul 07793, Republic of Korea). DNA samples (200 ng per sample) were processed according to the standard Axiom™ genotyping protocol. Briefly, genomic DNA was amplified, fragmented, and hybridized to the Korean Chip v2.0 arrays. Following hybridization, arrays were washed, stained, and scanned using the GeneTitan™ Multi-Channel Instrument (Thermo Fisher Scientific, Waltham, MA, USA).

#### 2.3.3. Quality Control

Raw intensity data were processed using Axiom Analysis Suite software (v5.1, Thermo Fisher Scientific). Quality control (QC) filtering was applied with the following criteria:

- Sample call rate ≥ 95%
- SNP call rate ≥ 95%
- Hardy–Weinberg equilibrium (HWE) *p*-value > 1 × 10^−6^ (noted for reference; not applicable for family data)
- Minor allele frequency (MAF) > 0.01 in the Korean reference population

All seven samples passed QC thresholds with call rates > 98%. Following QC, approximately 1.2 million high-quality biallelic SNPs were available for downstream analyses.

### 2.4. Candidate Gene Selection and Genetic Burden Score (GBS) Calculation

#### 2.4.1. AD Candidate Gene Selection

Six AD-associated genes were selected based on genome-wide significant associations (*p* < 5 × 10^−8^) in previous GWAS studies and functional relevance to AD pathology (Table 1).

#### 2.4.2. Variant Extraction and Annotation

SNPs located within candidate gene regions (±50 kb flanking regions) were extracted from the Korean Chip v2.0 genotype data. Risk allele designation was based on published genome-wide significant associations (*p* < 5 × 10^−8^) from the following sources:

- *APOE*: Kunkle et al. (2019) [16] GWAS meta-analysis (PMID: 30820047);
- *PICALM*, *CLU*, *CR1*, *BIN1*, and *ABCA7*: Bellenguez et al. (2022) [17] multi-ancestry GWAS (PMID: 35379992), Jansen et al. (2019) [18] meta-analysis (PMID: 30617256), and Lambert et al. (2013) [19] meta-analysis (PMID: 24162737);
- East Asian validation: Miyashita et al. (2023) [13] confirmed that risk allele directions for these six genes are concordant between European and East Asian populations (PMID: 35641666).

*p*-value 5. × 10^−8^ reported in the source GWAS publications cited above. The 320 SNPs included in the GBS calculation comprised the following:.

1. Lead GWAS SNPs (*n* = 2 for *APOE*: rs429358, rs7412): SNPs directly reported as genome-wide significant (*p* < 5 × 10^−8^) in source publications.
2. LD proxy variants (*n* = 318): SNPs in linkage disequilibrium (r^2^ > 0.6 in 1000 Genomes Project East Asian populations: JPT + CHB) with genome-wide significant lead variants. Risk allele designation for LD proxies was inferred based on the LD phase with the lead SNP’s risk allele.
3. Functional annotation filter: Among LD proxies, priority was given to variants with functional annotations in the Korean Chip v2.0 reference database (e.g., missense, regulatory, and eQTL effects).

All 320 SNPs trace back to genome-wide significant associations (*p* < 5 × 10^−8^) either directly (lead SNPs) or through established LD relationships (proxy SNPs). This approach ensures that risk allele designations are supported by robust statistical evidence while capturing Korean-specific haplotype structures.

Supplementary Table S2 provides a complete list of all 320 SNPs with rsIDs, genomic coordinates, risk allele designations, reference sources, and genotypes for all seven core family members.

#### 2.4.3. Variant Quality Control for GBS Calculation

Extracted variants underwent additional filtering to ensure high-quality risk allele assignment as follows:

- Biallelic SNPs only (multi-allelic variants excluded);
- Clear risk allele designation from published GWAS;
- Polymorphic within the family cohort (non-monomorphic sites);
- No missing genotypes across all seven members.

After filtering, 318 high-quality biallelic SNPs polymorphic within the seven core family members were identified from Korean Chip v2.0 genotyping, distributed across five candidate genes as follows: *ABCA7* (101 SNPs), *PICALM* (89 SNPs), *CR1* (84 SNPs), *CLU* (29 SNPs), and *BIN1* (8 SNPs).

Additionally, *APOE* genotypes at the two canonical ε-defining SNPs (rs429358 and rs7412) were determined independently (see Section 2.7), as these critical SNPs are not included in the Korean Chip v2.0 array. Combined with 7 additional *APOE* region variants from the array, the final GBS calculation included 320 SNPs total (*APOE*: 9 SNPs, including rs429358 and rs7412).

The distribution of variants across genes reflected both gene size and the density of polymorphic sites within this family.

#### 2.4.4. GBS Calculation

Genetic burden scores were calculated using an unweighted allele-counting approach.

We chose this method for several reasons appropriate to family-based studies. First, in familial contexts where shared genetic background and environment reduce confounding, within-family comparative analysis is more informative than absolute risk prediction based on population-derived effect sizes. Second, GWAS-derived effect sizes used in weighted polygenic scores are predominantly from European ancestry cohorts and may not optimally transfer to Korean families due to differences in allele frequencies and linkage disequilibrium patterns. Third, unweighted approaches avoid potential bias from external weights that may not reflect the genetic architecture of this specific family. While weighted polygenic scores may improve population-level prediction accuracy, our focus on within-family patterns prioritizes relative risk comparisons over absolute risk estimates.

For each individual i, GBS was computed as follows:

GBS_i = Σ(j = 1 to 320) G_ij

where G_ij represents the number of risk alleles (0, 1, or 2) for individual i at SNP j, summed across all 320 variants. Risk allele designation was based on published GWAS effect directions and functional annotations. Individual GBS values ranged from 37 to 61 risk alleles across the seven family members.

#### 2.4.5. Genetic Burden Score (GBS): Distinction from GWAS-Based Polygenic Risk Scores

We calculated a simplified genetic burden score (GBS) rather than a traditional genome-wide association study (GWAS)-derived polygenic risk score (PRS). This distinction is important, and we explicitly address it here to avoid confusion.

Traditional GWAS-Based PRS is as follows:

- Utilizes effect-size weights from large-scale GWAS (typically β coefficients or odds ratios);
- Includes 50,000 to >1 million genome-wide variants;
- Designed for population-level risk prediction;
- Requires external validation in independent cohorts;
- Output can be converted to disease probability or percentile rank;
- Assumes weights transfer across populations (often problematic for non-European ancestries).

Our GBS Approach is as follows:

- Uses simple unweighted allele counting (0, 1, or 2 risk alleles per SNP);
- Focuses on 320 variants across six well-established AD candidate genes (318 from Korean Chip v2.0 array + 2 *APOE* ε-defining SNPs genotyped independently);
- Designed for within-family relative comparison, not absolute prediction;
- No external weights applied (inappropriate for single-family studies);
- Output represents relative genetic burden within this family only;
- Cannot be compared to population PRS values or converted to disease probabilities.

Rationale for the GBS Approach in Family Studies is as follows:

1. Within-family comparison priority: Family studies aim to identify relative patterns among blood relatives sharing a genetic background, not to predict absolute disease risk. GBS is sufficient for comparing “high” vs. “low” burden siblings.
2. Population weight transferability issues: GWAS-derived effect sizes come from European ancestry cohorts. Applying these weights to Korean families may introduce bias due to differences in allele frequencies, linkage disequilibrium patterns, and gene–environment interactions.
3. Avoiding false precision: Using population-derived weights would imply prediction accuracy that cannot be validated in a single family. Unweighted GBS acknowledges the exploratory nature of the analysis.
4. Biological interpretability: Equal weighting allows direct interpretation of gene-specific contributions without confounding from external population parameters.

What Information May Be Missing:

By using a focused candidate-gene GBS (320 variants) rather than a genome-wide PRS (typically 50,000+ variants), we acknowledge the following limitations:

- Risk variants outside the six candidate genes (potentially 50+ additional GWAS loci);
- Rare family-specific variants with large effects (not captured on arrays);
- Structural variants and copy number variations;
- Protective variants that may explain resilience in high-GBS individuals;
- Gene–environment interactions;
- Epigenetic regulation of non-candidate genes.

Interpretation Boundaries:

- GBS values are NOT disease probabilities;
- GBS cannot be compared to population PRS percentiles (e.g., cannot say “this individual is in the top 10% of risk”);
- GBS is suitable ONLY for relative within-family comparison;
- Absolute GBS values have no clinical meaning outside this family context.

We use the term “Genetic Burden Score” rather than “Polygenic Risk Score” to clearly distinguish our method from weighted, genome-wide, population-validated PRS, while acknowledging that our analysis does involve multiple genetic loci (hence “polygenic” in the broad sense). Our GBS comprises 320 variants: 318 array-based SNPs plus rs429358 and rs7412 (*APOE* ε-defining SNPs not included in Korean Chip v2.0) (Supplementary Table S1 provides a detailed comparison of GBS vs. GWAS-based PRS features.)

### 2.5. DNA Methylation Analysis

#### 2.5.1. Peripheral Blood Methylation as a Proxy Biomarker

DNA methylation was assessed in peripheral blood samples. While brain tissue represents the primary site of AD pathology, blood-derived methylation patterns have been reported to correlate with certain brain methylation signatures and may serve as accessible biomarkers. However, several factors limit interpretation, as follows:

1. Tissue-specific regulation: Brain and blood cells have distinct epigenetic landscapes due to different developmental origins, environmental exposures, and regulatory mechanisms.
2. Cell-type composition: Peripheral blood consists of mixed cell populations (lymphocytes, monocytes, and granulocytes), each with unique methylation profiles. Bulk blood methylation represents an average across these cell types.
3. Uncertain concordance: Whether blood *APOE* methylation correlates with brain *APOE* methylation remains uncertain. Some studies report modest correlations (r = 0.3–0.5) for certain loci, while others show tissue-specific patterns with no cross-tissue concordance.
4. Systemic vs. local effects: Blood methylation may reflect systemic processes (inflammation, oxidative stress) rather than brain-specific AD pathology. Therefore, blood methylation should not be interpreted as directly reflecting brain epigenetic states, and our findings require validation in brain tissue before conclusions about AD pathophysiology can be drawn.

#### 2.5.2. Methylation Profiling Using Illumina EPICv2 Array

Genome-wide DNA methylation profiling was performed using the Illumina Infinium MethylationEPIC BeadChip v2 (EPICv2 array, Illumina Inc., San Diego, CA, USA), which interrogates over 930,000 CpG sites across the human genome with enhanced coverage of regulatory regions, CpG islands, shores, and shelves.

Methylation profiling services were provided by Macrogen Inc. (254 Beotkkot-ro, Geumcheon-gu, Seoul 08511, Republic of Korea). DNA samples (500 ng per sample, normalized to 50 ng/μL) were bisulfite-converted using the EZ DNA Methylation Kit (Zymo Research, Irvine, CA, USA) according to the manufacturer’s protocol. Bisulfite-converted DNA was amplified, fragmented, and hybridized to EPICv2 BeadChips overnight at 48 °C. Following hybridization, arrays were washed, stained, and scanned using the iScan System (Illumina Inc., San Diego, CA, USA)

#### 2.5.3. Methylation Data Pre-Processing and Quality Control

Raw intensity data (IDAT files) were processed using the R package minfi (v1.46.0). Pre-processing steps included the following:

- Background correction and normalization using the functional normalization (funnorm) method to adjust for technical variation;
- Probe filtering:
  ∗ Probes with detection *p*-value > 0.01 in any sample;
  ∗ Probes overlapping known SNPs (MAF > 0.01 in East Asian populations);
  ∗ Cross-reactive probes;
  ∗ Probes on sex chromosomes (for consistency across samples);
- Sample quality assessment: All seven samples exhibited high-quality metrics (median detection *p*-value < 0.001).

Beta values (β = methylated intensity/[methylated intensity + unmethylated intensity]) were calculated for each CpG site, representing methylation levels ranging from 0 (completely unmethylated) to 1 (fully methylated).

#### 2.5.4. Candidate Gene-Specific Methylation Analysis

For each of the six candidate genes, CpG probes located within gene bodies and regulatory regions (±10 kb) were extracted. Mean beta values across all CpG sites per gene were calculated for each individual to obtain gene-level methylation summaries. Gene-specific methylation levels were then correlated with corresponding SNP risk allele counts to assess genotype–methylation relationships.

#### 2.5.5. Functional Annotation and Clustering

Gene enrichment and functional annotation of CpG sites were conducted using the following:

- KYCG (Know Your CpGs) database (https://zhou-lab.github.io/sesame/dev/KYCG.html) (accessed via sesame package v1.18.0) (accessed on 15 December 2025) for CpG-centric functional annotations;
- gProfiler2 (v0.2.1) (https://biit.cs.ut.ee/gprofiler/) (accessed on 15 December 2025) for pathway enrichment analysis.

Hierarchical clustering of methylation profiles was performed using Euclidean distance and complete linkage methods to visualize overall epigenomic similarity among family members.

### 2.6. Statistical Analysis

#### 2.6.1. Descriptive Statistics

Continuous variables (GBS, methylation beta values, and clinical biomarkers) were summarized as mean ± standard deviation (SD) or median with interquartile range (IQR), as appropriate. Categorical variables (*APOE* genotype, dementia status) were presented as frequencies and percentages.

#### 2.6.2. Familial Aggregation and Generational Trends

Intraclass correlation coefficients (ICC) were calculated to assess familial clustering of GBS values among blood-related members. Generational trends in GBS were evaluated using linear regression, with generation coded as an ordinal variable (Generation I = 1, II = 2, III = 3).

#### 2.6.3. Gene-Gene and Genotype–Methylation Correlations

Pearson correlation coefficients were calculated to assess the following:

- Pairwise correlations between gene-specific risk allele counts (gene–gene interactions);
- Correlations between SNP risk allele counts and mean methylation beta values for each gene (genotype–methylation relationships).

Statistical significance was assessed at α = 0.05. Given the exploratory nature of this pilot study and small sample size (*n* = 7), correction for multiple testing was not applied. All statistical analyses should be interpreted with considerable caution due to fundamental limitations inherent to small sample sizes. Correlation coefficients may reflect sampling variation rather than true biological associations, and individual data points can substantially influence results. These analyses are intended strictly for hypothesis generation rather than definitive conclusions, and all findings require validation in larger, independent cohorts before drawing firm mechanistic or clinical inferences. The single affected individual in this cohort particularly limits our ability to establish generalizability or causality of observed associations.

#### 2.6.4. APOE ε4 Carrier Status Comparison

*APOE* ε4 carrier status was defined as possession of at least one ε4 allele (ε3/ε4 or ε4/ε4 genotypes). Within the seven core family members, total GBS distributions were compared between *APOE* ε4 carriers (*n* = 3) and non-carriers (*n* = 4) using descriptive statistics and visualizations.

### 2.7. APOE Genotype Determination

*APOE* genotypes were determined using direct genotyping of the two canonical *APOE* ε-defining SNPs (rs429358 and rs7412). As these SNPs are not included in the Korean Chip v2.0 array, *APOE* genotypes were independently determined via clinical genetic testing records and supplementary genotyping assays. Genotyping revealed four ε3/ε3 carriers (J-005, J-006, J-011, J-012) and three ε3/ε4 carriers (J-003, J-025, J-017). For the dementia patient (J-003), the *APOE* ε3/ε4 genotype was confirmed via independent clinical genetic testing records.

All genotypes were consistent with Mendelian inheritance patterns observed across three generations. For genetic burden score calculation, rs429358 and rs7412 were included alongside the 318 variants identified from the Korean Chip v2.0 genotyping, resulting in a total of 320 SNPs across six AD-associated genes (Supplementary Table S2).

### 2.8. Software and Data Visualization

All statistical analyses were performed using the following:

- R (v4.3.1, R Foundation for Statistical Computing, Vienna, Austria) for correlation analysis, ICC calculation, and methylation data processing;
- Python (v3.9.12, Python Software Foundation, Wilmington, DE, USA) for data manipulation and visualization:
  - pandas (v1.5.3) for data manipulation;
  - scipy (v1.10.1) for statistical tests;
  - matplotlib (v3.7.1) and seaborn (v0.12.2) for data visualization.
- PLINK (v1.9, https://www.cog-genomics.org/plink/, accessed on 20 January 2026) for genetic data quality control and file format conversion.

Pedigree diagrams were created using custom Python scripts. Correlation matrices, heatmaps, and radar plots were generated using seaborn and matplotlib. All figures were prepared at 300 DPI resolution, suitable for publication.

## 3. Results

### 3.1. Core Family Structure and Clinical Characteristics

**Figure 1:**
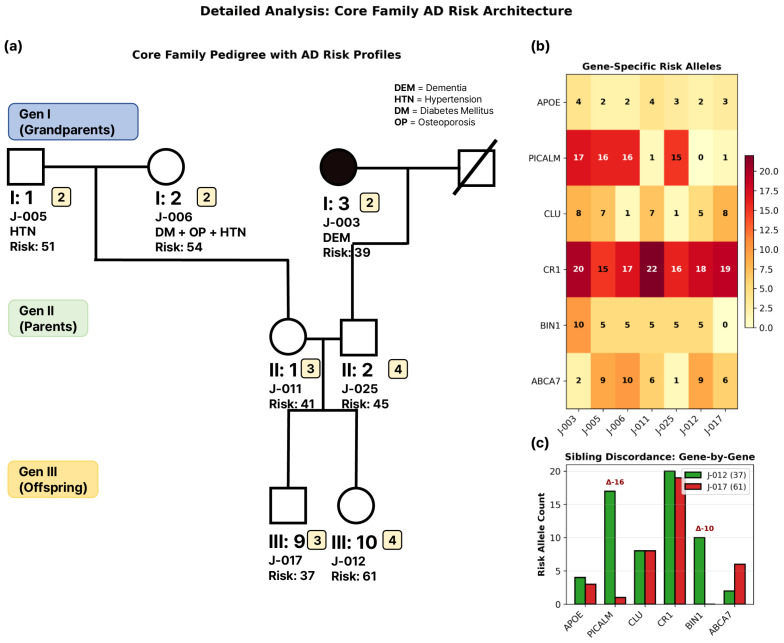
**Detailed Analysis of Core Family AD Risk Architecture Across Three Generations.** (**a**) Three-generation pedigree showing seven blood-related family members with genetic burden scores (GBS) and clinical phenotypes. Squares represent males, circles represent females, solid black fill indicates the affected proband, and diagonal lines indicate deceased individuals. Numbers inside symbols indicate *APOE* gene region risk allele count (from GBS calculation, not *APOE* ε4 carrier status). Individual J-003 (Generation I, proband) with dementia was observed to have the lowest GBS (39 alleles) while carrying the *APOE* ε3/ε4 genotype, while cognitively healthy grandparents show a higher burden (J-005: 51 alleles; J-006: 54 alleles). Generation III siblings display extreme discordance (J-012: 37 alleles; J-017: 61 alleles, Δ = 24). DEM = Dementia; HTN = Hypertension; DM = Diabetes Mellitus; OP = Osteoporosis. (**b**) Gene-specific risk allele heatmap across six AD-associated genes (*APOE*, *PICALM*, *CLU*, *CR1*, *BIN1*, *ABCA7*) for all seven family members. Color intensity represents allele count (yellow = low, red = high, scale 0–22 alleles). *CR1* contributes the highest burden across all individuals (15-22 alleles, mean 18.1), followed by PICALM (0–17 alleles, mean 9.4). *APOE* shows modest contribution (2–4 alleles, mean 2.9). (**c**) Sibling discordance analysis for Generation III brothers (J-012 and J-017). Green bars represent J-012 (GBS = 37); red bars represent J-017 (GBS = 61). The 24-allele difference is driven primarily by *CR1* (Δ = 16) and *PICALM* (Δ = 16), with smaller contributions from BIN1 (Δ = 10), *ABCA7* (Δ = 5), CLU (Δ = 3), and *APOE* (Δ = 1).

The study cohort comprised seven blood-related members across three generations: three grandparents (Generation I), two parents (Generation II), and two offspring (Generation III) (Figure 1a). One grandparent (J-003, female, *APOE* ε3/ε4) was clinically diagnosed with Alzheimer’s disease, while the remaining six members were cognitively normal. *APOE* genotypes included four ε3/ε3 carriers (J-005, J-006, J-011, and J-012) and three ε3/ε4 carriers (J-003, J-025, and J-017).

### 3.2. Genetic Burden Score Distribution and Generation I Pattern

GBS ranged from 37 to 61 alleles (mean 46.9 ± 8.8) across the seven family members (Figure 1a). Note: All quantitative values reported below are descriptive observations only; with *n* = 7 and one affected individual, no statistical inference or generalizability is possible. An unexpected pattern was observed in Generation I: the affected individual (J-003) exhibited the lowest GBS (39 alleles), while cognitively healthy grandparents showed substantially higher scores (J-005: 51 alleles; J-006: 54 alleles). (Descriptive pattern only; *n* = 7 precludes statistical inference) Generation II displayed intermediate values (mean 43.0 ± 2.8), while Generation III showed the widest range (37–61 alleles, mean 49.0 ± 17.0). Gene-specific analysis revealed that *CR1* contributed the most risk alleles across all individuals (15–22 alleles, mean 18.1, 38.6% of total GBS), followed by *PICALM* (0–17 alleles, mean 9.4, 20.0%) (Figure 1b). Despite its established role in AD, *APOE* contributed modestly (two to four risk alleles per individual from array-based variants, plus rs429358 and rs7412 genotypes, mean ~3 risk alleles, representing ~6% of total GBS). These counts represent *APOE* gene region SNPs, not simply *APOE* ε4 carrier status. Notably, the three *APOE* ε4 carriers (J-003, J-025, and J-017) exhibited a 22-allele range in total GBS (39–61), demonstrating that *APOE* ε4 carrier status does not predict overall genetic burden in this family. The dementia patient J-003 carried only two *APOE* risk alleles, equal to J-005 and J-006 (both healthy grandparents with two alleles each), suggesting that neither *APOE* ε4 carrier status nor *APOE* gene region risk allele burden alone corresponded with disease phenotype in this family. However, with only one affected individual, this observation requires validation in larger familial cohorts.

### 3.3. Extreme Sibling Discordance in Generation III

Generation III siblings exhibited a 24-allele difference (J-012: 37 vs. J-017: 61, 64.9% relative difference), the largest discordance in the family (Figure 1c). (Descriptive observation; stochastic segregation in *n* = 2 siblings) This difference was primarily driven by *CR1* (Δ = 16 alleles) and *PICALM* (Δ = 16 alleles), while other genes showed smaller variations (*BIN1*: Δ = 10; *ABCA7*: Δ = 5; *CLU*: Δ = 3; *APOE*: Δ = 1). Notably, J-012 inherited fewer risk alleles than either parent, while J-017 accumulated substantially more, illustrating the stochastic nature of polygenic inheritance.

### 3.4. APOE ε4 Carrier Status and Genetic Burden

**Figure 2:**
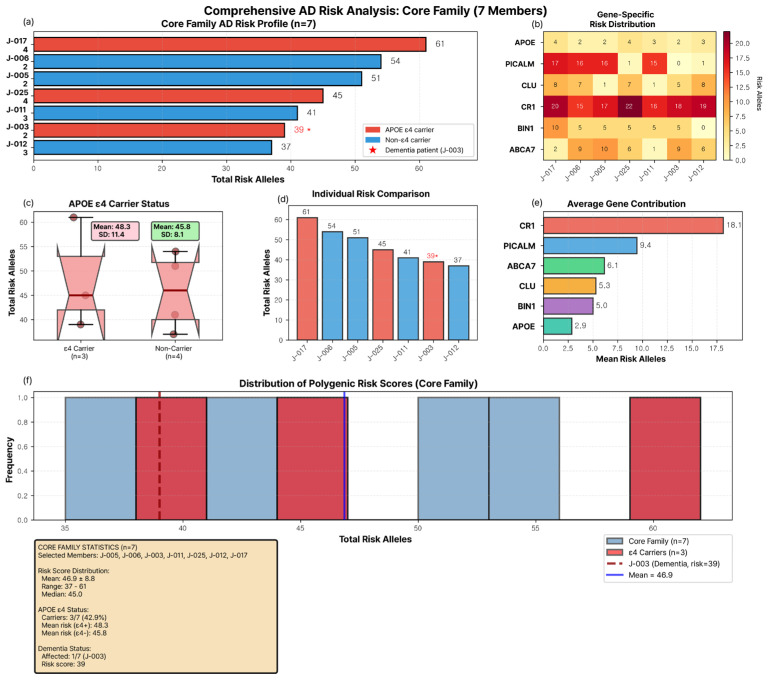
**Comprehensive Genetic Burden Score Analysis of the Core Family.** (**a**) Total GBS distribution ordered by burden. Red bars = *APOE* ε4 carriers (*n* = 3: J-003, J-025, J-017, all ε3/ε4), blue bars = non-carriers (*n* = 4: all ε3/ε3). The three ε4 carriers span the entire GBS spectrum: J-003 (dementia, lowest GBS = 39), J-025 (healthy, GBS = 45), and J-017 (healthy, highest GBS = 61), demonstrating that APOE ε4 carrier status does not predict overall genetic burden in this family. (**b**) Gene-specific risk allele heatmap across six AD genes. *CR1* dominates (15–22 alleles per individual), while *APOE* contributes minimally (2–4 alleles from array-based variants; note that this count represents *APOE* gene region *SNPs*, not *APOE* ε4 allele presence). Color scale: yellow (low) to red (high). (**c**) Box plot comparing GBS between ε4 carriers (mean 48.3 ± 11.4) and non-carriers (mean 45.8 ± 8.1). The minimal difference indicates a weak association between *APOE* ε4 status and total genetic burden. (**d**) Individual risk comparison showing a substantial difference between the lowest (J-012, 37) and the highest (J-017, 61) risk members. Red asterisk (*) indicates the affected individual (J-003) with intermediate GBS (39). (**e**) Average gene contribution: *CR1* (18.1, 38.6%), *PICALM* (9.4, 20.0%), *ABCA7* (6.1, 13.0%), *CLU* (5.3, 11.3%), *BIN1* (5.0, 10.7%), *APOE* (2.9, 6.2%). (**f**) GBS distribution histogram. Light blue = all members, red = ε4 carriers. The dashed line indicates J-003 (39), the solid line indicates the mean (46.9).

*APOE* ε4 carriers (*n* = 3: J-003, J-025, J-017) demonstrated only marginally higher mean GBS compared to non-carriers (*n* = 4) who showed similar mean GBS (Figure 2a,c). (Descriptive comparison only; *n* = 3 vs. *n* = 4 precludes statistical testing) Box plot analysis revealed substantial overlap in GBS distributions between the two groups, with ε4 carriers spanning the entire risk spectrum from 39 to 61 alleles (Figure 2c). The distribution histogram further illustrated this pattern (Figure 2f). While ε4 carriers (red bars) were distributed across multiple risk strata, the dementia patient J-003 (dashed line at 39 alleles) fell substantially below the family mean (solid line at 46.9 alleles). Conversely, two non-ε4 carriers (J-005: 51; J-006: 54) exceeded the mean, and the highest-risk individual (J-017, 61 alleles, ε4 carrier) remained cognitively healthy (Figure 2a,d). This distribution pattern in this single family suggested that *APOE* ε4 carrier status did not directly correspond with individual genetic burden or disease phenotype, though the single affected individual limits interpretation.

### 3.5. Gene-Specific Contributions to Family-Wide Genetic Burden

Analysis of average gene contributions across all seven members revealed a consistent hierarchy (Figure 2e). *CR1* contributed the most risk alleles (mean 18.1, 38.6% of total GBS), followed by *PICALM* (9.4, 20.0%), *ABCA7* (6.1, 13.0%), *CLU* (5.3, 11.3%), *BIN1* (5.0, 10.7%), and *APOE* (2.9, 6.2%). This pattern remained stable across all individuals despite substantial variation in total GBS (Figure 2b). The dominance of *CR1* and *PICALM*, collectively accounting for 58.6% of total risk alleles, suggested that these genes play central roles in this family’s AD genetic architecture. In contrast, *APOE*’s modest contribution (6.2%) suggested that its influence on disease risk may operate through mechanisms beyond simple allele burden, potentially involving gene expression or epigenetic regulation.

### 3.6. Gene–Gene Correlations and Individual Risk Profiles

**Figure 3:**
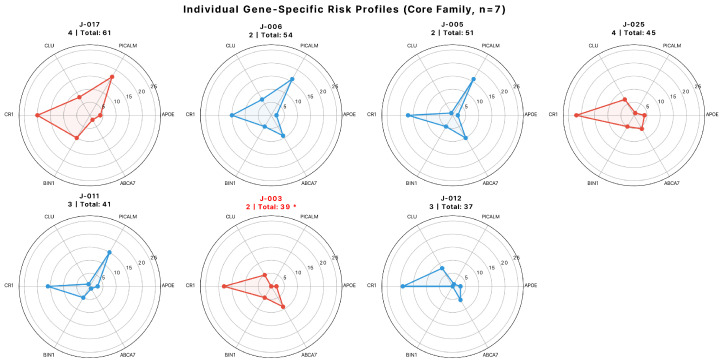
**Individual Gene-Specific Risk Profiles (Core Family, *n* = 7).** Radar plots showing gene-specific risk allele distributions for all seven family members. Each axis represents one gene (*APOE*, *PICALM*, *CLU*, *CR1*, *BIN1*, and *ABCA7*) with scales from 0 to 25 alleles. Red plots indicate *APOE* ε4 carriers (J-003, J-017, and J-025); blue plots indicate non-carriers (J-005, J-006, J-011, and J-012). The affected individual J-003 (red plot, marked with asterisk *) shows an elevated *CR1* burden (18 alleles) but a complete absence of *PICALM* risk alleles (0 alleles), a profile distinct from all other family members. The highest-risk individual, J-017, shows maximum *CR1* (20 alleles) and *PICALM* (17 alleles). No two individuals share identical gene-specific configurations, illustrating the complexity of polygenic inheritance even within a single family.

Individual gene-specific risk profiles revealed substantial heterogeneity in polygenic architecture (Figure 3). Despite carrying the lowest total GBS (39 alleles), the affected individual J-003 showed elevated *CR1* burden (18 alleles) but complete absence of *PICALM* risk alleles (0 alleles), a profile distinct from all other family members. Note: We initially calculated pairwise correlations between gene-specific risk allele counts. However, with only seven observations (*n* = 7), correlation coefficients are highly unstable—confidence intervals span ±0.4 to ±0.6, and a single data point can substantially alter results. We have therefore moved this analysis to Supplementary Figure S1 with extensive caveats and do NOT interpret these patterns as evidence for biological mechanisms. All correlation values should be considered illustrative only and require validation in larger cohorts. No two individuals shared identical gene-specific configurations, illustrating the complexity of polygenic inheritance even within a single family. For example, Generation I grandparents J-005 and J-006, despite comparable total risk (51 vs. 54 alleles), displayed divergent patterns: J-005 showed high *PICALM* (16) with moderate *CR1* (15), while J-006 exhibited similar *PICALM* (16) but higher *ABCA7* (9 vs. 2). This inter-individual variability suggested that total GBS alone may obscure important gene-specific differences that could influence disease susceptibility.

### 3.7. Intergenerational Patterns of Genetic Burden

**Figure 4:**
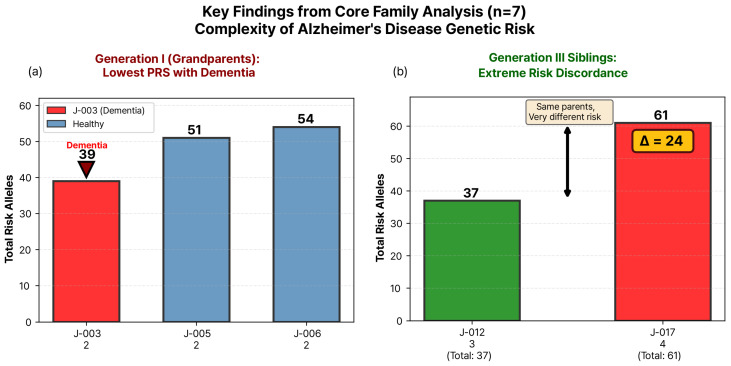
**Key Observations Highlighting the Complexity of AD Genetic Risk.** (**a**) Generation I pattern showing affected individual J-003 (red bar) with the lowest GBS (39 alleles), while cognitively healthy grandparents show substantially higher burden (J-005: 51 alleles; J-006: 54 alleles). Numbers below bars indicate *APOE* gene region risk allele counts (from GBS calculation). (**b**) Extreme sibling discordance in Generation III showing brothers J-012 (green, 37 alleles) and J-017 (red, 61 alleles) with a 24-allele difference (Δ = 24) despite sharing identical parents. Numbers below indicate *APOE* gene region risk allele counts (3 vs. 4).

Analysis of GBS across three generations revealed distinct patterns in burden distribution (Supplementary Figure S2). Note: Small sample size per generation (*n* = 2–3) precludes statistical trend testing. Patterns shown are descriptive only and may reflect stochastic variation inherent to small samples rather than meaningful biological trends. Key observations are illustrated in Figure 4: the affected individual J-003 exhibited the lowest GBS (39 alleles) among Generation I grandparents (Figure 4a), while Generation III siblings displayed extreme discordance (Δ = 24 alleles, Figure 4b) despite sharing identical parents. Generation I (grandparents, *n* = 3) exhibited a mean GBS of 48.0 ± 7.9 (range: 39–54), with the affected individual J-003 representing the lowest value despite clinical disease manifestation. Generation II (parents, *n* = 2) displayed the most stable and lowest mean GBS (43.0 ± 2.8, range: 41–45), suggesting potential regression toward lower genetic burden. In contrast, Generation III (offspring, *n* = 2) showed the highest mean GBS (49.0 ± 17.0, range: 37–61) with dramatically increased variability, reflecting the stochastic nature of allele segregation during meiotic recombination. Gene-specific contribution analysis revealed both stable and dynamic patterns across generations (Supplementary Figure S2). *CR1* maintained its dominance throughout all three generations, contributing 16.7, 19.0, and 19.5 alleles to Gen I, II, and III, respectively. *PICALM* showed a declining trend from Gen I (10.7 alleles) to Gen II (8.0), with modest recovery in Gen III (9.0). Conversely, *CLU* exhibited dramatic expansion in Gen III (8.0 alleles), nearly doubling its contribution compared to earlier generations (Gen I: 4.3; Gen II: 4.0). *APOE* contribution remained stable and modest across all generations (2.0–3.5 alleles), while *BIN1* and *ABCA7* showed intermediate, relatively stable contributions. The generational mean values illustrated a V-shaped pattern, with the middle generation exhibiting reduced genetic burden followed by a rebound in the offspring generation, likely driven by extreme sibling discordance in Gen III (Δ = 24 alleles between J-012 and J-017).

### 3.8. APOE Methylation Patterns in Relation to Genetic Burden

**Figure 5:**
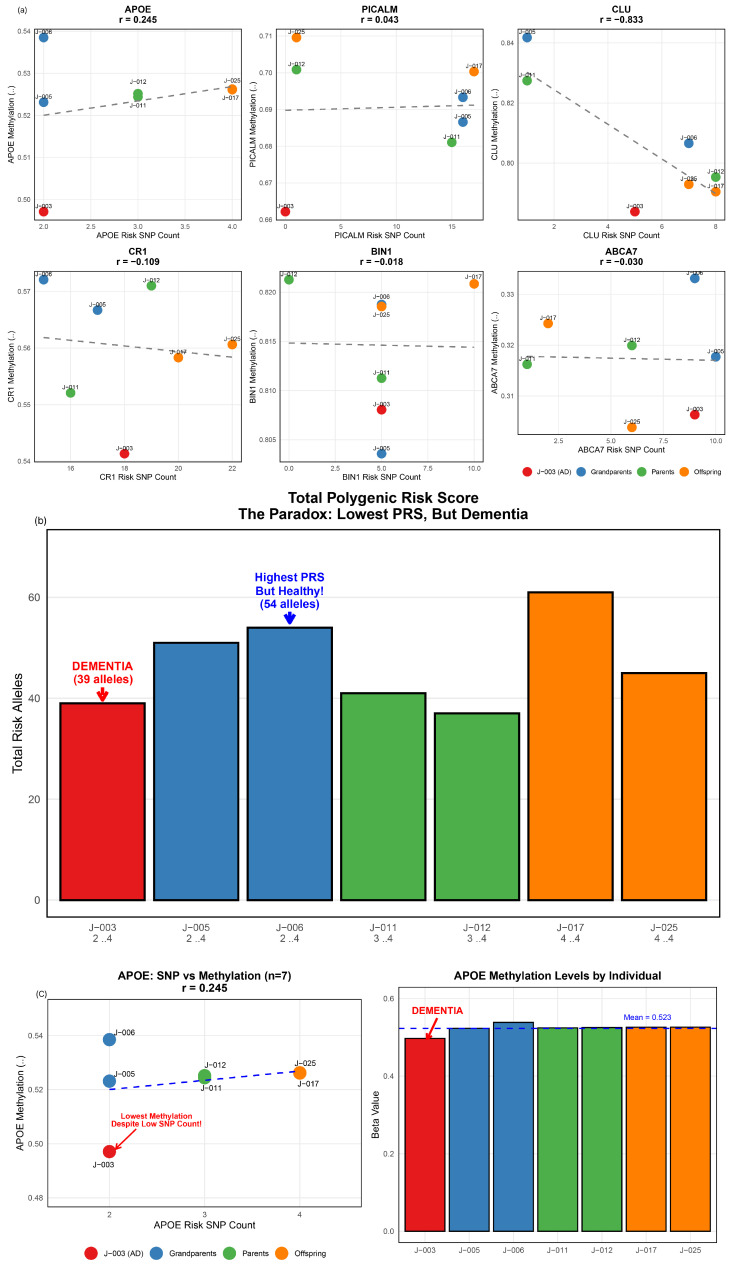
***APOE* Methylation Patterns in Relation to Genetic Burden.** (**a**) Correlation between risk SNP counts and methylation levels for six AD genes. Dashed lines represent linear regression trend lines. *CLU* shows a strong negative correlation (r = −0.833); however, with only seven observations, this correlation is highly unstable and should not be interpreted as evidence for biological mechanisms. *APOE* shows a weak positive correlation (r = 0.245). *PICALM* (r = 0.043), *CR1* (r = −0.109), *BIN1* (r = −0.018), and *ABCA7* (r = −0.030) show negligible correlations. The affected individual, J-003 (red), exhibited the lowest *APOE* methylation despite similar SNP counts to healthy grandparents. (**b**) Total GBS comparison showing J-003 (affected, red, 39 alleles) with the lowest genetic burden, while J-006 (healthy, blue, 54 alleles) has the highest. Numbers below bars indicate *APOE* gene region risk allele counts (2–4 alleles, representing SNP burden, not ε4 allele count). (**c**) *APOE* methylation levels across seven individuals. J-003 exhibited the lowest methylation (β = 0.495), below the family mean (β = 0.523, dashed line), while other members clustered near or above the mean (range: 0.520–0.540).

To investigate potential epigenetic patterns, we examined the relationship between genetic variants and DNA methylation across all six candidate genes (Figure 5a). Among the six genes analyzed, *CLU* showed a negative correlation between SNP count and methylation (r = −0.833). However, with only seven observations, this correlation is highly unstable and should not be interpreted as evidence for biological mechanisms. *APOE* showed a weak positive correlation (r = 0.245), while *PICALM* (r = 0.043), *CR1* (r = −0.109), *BIN1* (r = −0.018), and *ABCA7* (r = −0.030) exhibited negligible associations between SNP count and methylation. Individual-level analysis revealed that the affected individual J-003 exhibited the lowest *APOE* methylation level (β = 0.495) among all family members (Figure 5c), below the family mean (β = 0.523). Notably, J-003 shared identical *APOE* SNP counts (2 alleles) with two healthy grandparents (J-005: β = 0.520; J-006: β = 0.540), yet displayed lower methylation.

Three Possible Interpretations: With only one affected individual and cross-sectional data, we cannot determine which of the following scenarios applies: (1) Causal pathway hypothesis: *APOE* hypomethylation preceded disease onset and contributed to AD pathogenesis through increased *APOE* expression. Previous studies have reported that DNA hypomethylation at gene promoters can influence transcriptional activity and that *APOE* expression levels have been associated with amyloid-β metabolism. (2) Disease consequence hypothesis: Methylation decreased as a result of disease-related processes such as neuroinflammation, oxidative stress, or treatment effects. Studies have shown that these factors present in AD brains can alter DNA methylation patterns in peripheral blood. (3) Unrelated biological variation: The observed methylation difference falls within normal inter-individual methylation variation and is coincidentally associated with disease status in this single family. Distinguishing between these scenarios requires longitudinal methylation assessment in multiple at-risk individuals before and after disease onset, which is beyond the scope of this single-family cross-sectional study.

Technical Considerations: Several technical factors limit interpretation of the *APOE* methylation difference: 1. Magnitude of difference: The methylation difference between J-003 (β = 0.495) and family mean (β = 0.523) is modest. Illumina EPIC arrays have technical variability of ~2–5%, meaning the observed difference approaches the assay’s precision limits. 2. No external reference: We do not have healthy control data from age-matched Korean individuals without a family AD history. Therefore, we cannot assess whether J-003’s methylation level is “pathologically low” or whether the other family members have “unusually high” methylation. 3. Batch effects: All samples were processed in a single batch. Batch-related technical variation cannot be assessed or excluded. 4. Peripheral blood measurement: Methylation was measured in peripheral blood, not brain tissue. Whether blood *APOE* methylation correlates with brain methylation patterns remains uncertain. 5. Cell-type composition: Blood methylation reflects mixed cell populations (lymphocytes, monocytes, granulocytes). Cell-type-specific signals may be diluted or confounded. These technical limitations preclude definitive interpretation of the methylation difference as biologically or clinically meaningful.

## 4. Discussion

This exploratory single-family case study observed an unexpected pattern that raises fundamental questions about personalized risk prediction in Alzheimer’s disease: the individual with dementia had the lowest genetic burden score (39 alleles), while those with substantially higher burdens (51–61 alleles) remained cognitively healthy [20,21]. This observation highlights a critical challenge in precision medicine—translating population-level genetic associations into accurate individual-level risk assessments. Notably, the affected individual also exhibited lower *APOE* DNA methylation levels (β = 0.495) compared to unaffected members [22]. However, with only one affected individual, this observation cannot establish causality, directionality, or generalizability. Rather, it generates the hypothesis that epigenetic factors may relate to phenotypic variability not fully captured by genetic burden scores alone-a hypothesis requiring rigorous testing in substantially larger cohorts before any mechanistic or clinical implications can be inferred [23,24]. The most notable observation was the inverse relationship between genetic burden and disease phenotype observed in Generation I. Individual J-003, carrying only 39 risk alleles—the lowest in the family—developed Alzheimer’s disease, while two grandparents with 30–38% higher genetic burden remained cognitively normal into advanced age [25]. This pattern appears inconsistent with the typical expectation that accumulated risk alleles correlate positively with disease probability [26]. However, with only one affected individual, this observation may reflect unique circumstances specific to this person rather than a generalizable biological pattern. Similar unexpected patterns have been reported in other complex diseases, including coronary artery disease and type 2 diabetes, though typically attributed to environmental factors or measurement variability [27]. However, the controlled genetic background of family studies eliminates population stratification and reduces environmental heterogeneity, making the paradox observed here particularly notable [28]. Previous family-based AD studies identified *APOE* ε4 carriers remaining cognitively intact into their 90 s while ε3/ε3 individuals developed early dementia, but lacked the multi-omics framework necessary to identify mechanistic explanations [29]. Our integration of methylation data provides a preliminary molecular context for understanding the relationship between genetic burden and phenotypic outcome [30]. Beyond genetic analysis, epigenetic profiling revealed that the affected individual J-003 exhibited lower *APOE* methylation levels (β = 0.495), below the family mean, falling below all other members who clustered tightly around β = 0.523. Notably, J-003 shared identical *APOE* gene region SNP counts (two alleles) with two healthy grandparents (J-005: β = 0.520, J-006: β = 0.540), yet displayed lower methylation. Causality Cannot Be Established: The cross-sectional design and single affected individual preclude determining whether *APOE* hypomethylation. Preceded disease onset and contributed to pathogenesis (etiological factor). Decreased as a consequence of disease-related inflammation, oxidative stress, or medication (epiphenomenon). Represents normal biological variation unrelated to disease (a coincidental finding). Importantly, the consequence hypothesis is equally plausible to the causal hypothesis.

Previous studies have shown that neuroinflammation and oxidative stress—both present in AD brains—can alter DNA methylation patterns in peripheral blood [8]. Therefore, the methylation difference observed here may be a consequence rather than a cause of disease. Requirements for Causal Inference: Distinguishing between these scenarios requires longitudinal methylation tracking BEFORE symptom onset in multiple at-risk individuals. Assessment of conversion vs. non-conversion in relation to baseline methylation. Functional experiments demonstrating methylation-to-phenotype causality. Brain tissue validation confirming peripheral-central concordance. None of these requirements is met in the current study. However, this observation generates the hypothesis that epigenetic regulation may vary independently of genetic variation in familial contexts, warranting investigation in larger cohorts [8]. Previous studies have reported that DNA hypomethylation at gene promoters can influence transcriptional activity and that elevated *APOE* expression—particularly of the ε4 isoform—has been associated with impaired amyloid-β clearance and tau pathology [30]. Whether the lower *APOE* methylation observed in J-003 plays a causal role in disease pathogenesis, represents a consequence of neurodegeneration, or reflects unrelated biological variation cannot be determined from this study design [31]. The observed negative correlation between *CLU* risk alleles and methylation (r = −0.833), while notable, is based on seven data points and is highly unstable. A single individual’s data can substantially alter this correlation. This pattern requires validation in larger cohorts before any biological interpretation. Functional studies in larger cohorts, ideally with longitudinal methylation assessments before and after disease onset, are essential to test whether epigenetic dysregulation relates to disease risk or represents a biomarker of other processes [32]. The observation that J-003 exhibited both low genetic burden and low *APOE* methylation suggests the hypothesis that epigenetic patterns may contribute to phenotypic variability in familial contexts, though alternative explanations (consequence, coincidence) remain equally plausible [33]. The broad gene–gene correlation patterns revealed by our analysis suggest that polygenic risk is not specific. Analysis revealed that *CR1*, not *APOE*, dominated genetic burden in this family, with *APOE* ε4 carriers differing from non-carriers by only 2.6 alleles [34]. This apparent distinction reflects the difference between effect size and allele burden: *APOE* ε4 is low-frequency, high-impact (MAF ~10–15%), while *CR1* harbors numerous common variants contributing many alleles with modest individual effects [17]. These findings suggest that *APOE* may influence disease through multiple layers—genotype, allele burden, and methylation—all potentially requiring consideration for individual assessment, though this requires validation [35]. The extreme sibling discordance observed in Generation III (24-allele GBS difference) illustrates the stochastic and unpredictable nature of polygenic inheritance. Despite sharing identical parents and similar environments, brothers J-012 and J-017 differed by 24 risk alleles, driven primarily by differences in *CR1* (Δ = 16) and *PICALM* (Δ = 16). Independent assortment during meiosis randomly distributes parental alleles, creating extreme configurations wherein offspring can exhibit substantially lower or higher burden than either parent. (Note: *n* = 2 siblings limits the generalizability of this pattern.) The V-shaped generational pattern (Gen I: 48 → Gen II: 43 → Gen III: 49) may reflect both regression toward the mean in Generation II and variance expansion in Generation III (SD = 17.0, six-fold higher than Gen II), though with only 2–3 individuals per generation, this pattern should be interpreted cautiously [36]. Gene-specific dynamics revealed that while *CR1* maintained stable dominance (16.7–19.5 alleles) across generations, *CLU* nearly doubled in Generation III (8.0 vs. 4.0–4.3), indicating that specific risk haplotypes can emerge through recombination. Collectively, these patterns suggest that genetic burden evolves dynamically across generations, with different genes showing distinct inheritance trajectories that cannot be predicted from parental profiles alone [37]. This study has fundamental limitations that constrain interpretation. Most critically, the cohort includes only one affected individual, which fundamentally precludes establishing causality, generalizability, or the direction of observed associations. The presence of only one affected individual fundamentally constrains all interpretations: we cannot establish causality or directionality, cannot assess sensitivity or specificity of any finding, cannot evaluate concordance patterns, and cannot control for individual-specific factors such as lifestyle, comorbidities, environmental exposures, or treatment history. Any observed difference could reflect the unique circumstances of this individual rather than generalizable mechanisms. This limitation cannot be overstated: patterns observed in a single affected individual may reflect unique circumstances specific to that person rather than generalizable biological mechanisms. This limitation cannot be mitigated through statistical methods or analytical sophistication—it requires replication in independent families with multiple affected individuals. All findings should be considered strictly hypothesis-generating. Validation in substantially larger familial cohorts with multiple affected individuals is required before any mechanistic or clinical inferences can be drawn [38]. The lower *APOE* methylation in J-003 permits three equally plausible interpretations. First, hypomethylation may have preceded AD onset and contributed to pathogenesis through increased *APOE* expression, enhanced Aβ production, or impaired clearance (risk factor hypothesis). Second, methylation may have decreased as a result of neuroinflammation (systemic cytokine effects on blood methylation), oxidative stress (reactive oxygen species altering DNMT activity), disease duration (progressive epigenetic dysregulation), medication effects (cholinesterase inhibitors, memantine), or age-related drift accelerated in disease states (consequence hypothesis). Evidence supporting this consequence hypothesis includes the fact that neuroinflammation in AD brains releases systemic cytokines that alter DNA methyltransferase activity in peripheral blood, oxidative stress markers, and correlate with global DNA hypomethylation in AD patients, and longitudinal studies show methylation changes following cognitive decline, not preceding it. Third, the observed methylation difference may fall within the normal biological variation range and be unrelated to disease (coincidence hypothesis). J-003 may have constitutively lower *APOE* methylation due to genetic variants affecting methylation (meQTLs), environmental exposures (diet, smoking, and stress), stochastic epigenetic drift, or technical measurement variability. With single-timepoint, single-individual data, we cannot distinguish between these scenarios. All three remain equally plausible given the available evidence. Importantly, we cannot exclude—and indeed consider it likely—that J-003’s lower *APOE* methylation is a result of her disease rather than a contributor to it. To establish the causal scenario would require longitudinal pre-symptomatic methylation tracking showing hypomethylation precedes cognitive decline by years in multiple at-risk individuals. Establishing the consequence scenario would require a cross-sectional comparison showing that methylation correlates with disease severity, duration, or inflammatory markers across multiple affected individuals. To establish the coincidence scenario would require large population studies showing such methylation variation is common and uncorrelated with AD risk. None of these validations are possible with our current data. Furthermore, the correlations reported in this study (e.g., gene–gene correlations, *CLU* methylation: r = −0.833) are based on only seven data points. In samples this small, correlation coefficients are highly unstable, and a single outlier can drive the entire association [39]. These values should not be interpreted as reliable effect size estimates but rather as preliminary observations requiring validation in appropriately powered studies. For example, a correlation of r = 0.78 based on *n* = 7 has a 95% confidence interval of approximately [−0.15, 0.96], meaning the true correlation could be near zero, moderate, or strong—the point estimate alone is misleading. Changing a single individual’s value by 2–3 alleles could reverse the direction of several correlations. Similarly, generational patterns (V-shaped trend, sibling discordance) may reflect stochastic variation inherent to small samples rather than meaningful biological signals. All findings should be considered strictly hypothesis-generating and require validation in larger familial cohorts and independent populations before drawing firm mechanistic conclusions [40]. DNA methylation was measured in peripheral blood rather than brain tissue, the primary site of AD pathology [41]. This is a fundamental limitation that constrains interpretation. meta-analysis of AD brain methylation [31] found 3517 differentially methylated loci, of which only approximately 30% showed concordant changes in blood. *APOE* methylation specifically shows modest brain–blood correlation (r = 0.35–0.45) in some studies but tissue-specific patterns in others. Cell-type-specific analysis reveals that neuronal *APOE* methylation differs from glial, endothelial, and blood cell methylation. This means J-003’s lower blood *APOE* methylation may not reflect her brain *APOE* methylation. Even if blood-brain methylation correlates on average, individual-level concordance may be poor. Blood-based methylation may not fully reflect brain-specific epigenetic states. While *APOE* is systemically expressed and peripheral dysregulation may correlate with central effects, this remains uncertain. Blood methylation findings require validation in postmortem brain tissue or cerebrospinal fluid-derived cells [42]. Third, the cross-sectional design prevents assessment of temporal dynamics and causality. Whether *APOE* hypomethylation precedes clinical symptoms (suggesting potential causal involvement) or arises as a consequence of disease processes cannot be determined from this study. Longitudinal studies tracking methylation trajectories before and during AD progression would be essential to address causality. Environmental factors known to influence methylation—diet, physical activity, and stress—were not systematically assessed and may contribute to observed patterns [43]. Individual-specific exposures (smoking history, dietary folate intake, physical activity levels, and psychosocial stress) could explain methylation differences independent of disease status. Lastly, our candidate–gene approach focused on six well-established loci, potentially missing genome-wide genetic contributions. By using a focused candidate–gene GBS rather than genome-wide PRS, we potentially miss approximately 50 or more additional GWAS-identified AD risk loci, rare variants with large effects (MAF less than 1%), protective variants that may explain resilience in high-GBS individuals, family-specific rare mutations, gene–environment interactions, and structural variants and copy number variations. The 6-gene GBS likely captures only 30–40% of heritable genetic risk, based on GWAS variance explained estimates. The “missing heritability” could explain why high-GBS individuals remain healthy—they may carry protective variants outside our analyzed genes. Recent studies show that even with thousands of variants, marginal contributions become vanishingly small, though this does not negate the potential importance of unmeasured variants in individual cases. If validated in substantially larger cohorts, these findings may suggest potential directions for future research. DNA methylation profiling—particularly at *APOE*—could potentially complement genetic burden assessment to identify individuals with unfavorable epigenetic patterns despite reassuring genetic profiles. However, this requires validation in cohorts with multiple affected individuals and longitudinal follow-up before any clinical application can be considered. Unlike immutable genetic variants, methylation patterns are potentially modifiable through pharmacological or lifestyle interventions, though this requires extensive validation [44]. However, the gene-specific correlation observed for *CLU* (though based on only *n* = 7 and thus unstable) suggests that global methylation changes may have unintended consequences, necessitating locus-specific approaches if this avenue is pursued. Family-based genetic counseling should emphasize the unpredictability of polygenic inheritance and the necessity of individual genotyping rather than reliance on parental burden estimates, as siblings can differ substantially in genetic burden despite identical parentage [45]. *CR1*’s dominance over *APOE* in this family suggests that gene-specific burden profiles—not just total scores—may be relevant for understanding individual risk patterns, though this requires validation. If validated in substantially larger cohorts, gene-specific profiling could inform future therapeutic development targeting specific pathways (e.g., complement modulation for *CR1*-driven risk, endocytic enhancement for *PICALM*-driven risk). However, current findings are insufficient for clinical recommendations, and extensive validation is required before any therapeutic implications can be considered [33].

Future studies should expand to larger multi-generational families to enable robust heritability estimation and statistical inference. Longitudinal follow-up of high-burden but currently unaffected individuals (particularly J-017, GBS = 61) would test whether elevated genetic burden predicts future conversion or whether protective factors maintain resilience [46]. Functional validation of *APOE* hypomethylation using patient-derived iPSCs differentiated into neurons, combined with CRISPR-based epigenome editing, could establish whether methylation causally affects *APOE* expression and downstream pathology. However, such studies are preliminary steps in a decades-long validation pathway and do not imply clinical applicability. The integration of transcriptomics, proteomics, and metabolomics would provide comprehensive molecular profiles, while meQTL mapping could clarify genetic influences on methylation patterns [47]. Extension to diverse ancestries is essential to assess whether genetic–epigenetic interactions represent universal mechanisms or population-specific phenomena [48].

Critical Limitations That Preclude Hypothesis Generation: While this study demonstrates the technical feasibility of integrated genomic-epigenomic profiling, several fundamental limitations prevent the formulation of testable hypotheses: 1. Single affected individual: Patterns observed in one person may reflect unique circumstances (specific environmental exposures, comorbidities, medication history, and stochastic biological variation) rather than generalizable mechanisms. 2. Cross-sectional design: Even if the methylation difference were replicated in larger cohorts, temporal directionality (cause vs. consequence) cannot be inferred without longitudinal pre-symptomatic assessment. 3. Unmeasured confounders: Numerous factors not assessed in this study (diet, physical activity, stress, inflammation markers, other medications, and smoking history) could explain both the methylation pattern and disease outcome. 4. Tissue mismatch: Blood methylation may not reflect brain epigenetic states, the primary site of AD pathology. 5. Technical variability: The modest methylation difference (Δβ = 0.028) approaches the technical precision limits of the Illumina EPIC array (~2–5% variability). Therefore, while our observations are consistent with multiple plausible biological scenarios, they do not constitute evidence sufficient to prioritize any specific hypothesis for further investigation. Replication in independent families with multiple affected individuals is the essential first step before mechanistic pathways can be explored.

In conclusion, this exploratory single-family case study observed an unexpected pattern where the individual with dementia had the lowest genetic burden score (39 alleles) while higher-burden individuals (51–61 alleles) remained cognitively healthy. The affected individual also exhibited lower APOE methylation (β = 0.495) compared to the family mean (β = 0.523). However, with only one affected individual, no conclusions can be drawn regarding causality, generalizability, or the biological significance of these observations. The methylation difference may represent a causal factor preceding disease onset, a consequence of disease-related processes (neuroinflammation, oxidative stress), or coincidental biological variation unrelated to disease—scenarios that cannot be distinguished with cross-sectional data from a single case. Several fundamental limitations preclude hypothesis generation from the following dataset: (1) patterns observed in one affected person may reflect unique individual circumstances rather than generalizable mechanisms; (2) temporal directionality (cause versus consequence) cannot be inferred without longitudinal pre-symptomatic assessment; (3) numerous unmeasured confounders (lifestyle factors, comorbidities, medications) could explain the observed patterns; (4) peripheral blood methylation may not reflect brain epigenetic states; and (5) the modest methylation difference (Δβ = 0.028) approaches the technical precision limits of the array platform. These descriptive observations demonstrate the technical feasibility of integrated genomic-epigenomic profiling in Korean families using population-specific platforms (Korean Chip v2.0, Illumina EPICv2). The study serves as proof-of-concept for the methodology rather than as evidence for specific biological mechanisms. Validation in substantially larger cohorts with multiple affected individuals across multiple families, longitudinal methylation tracking before and after disease onset, functional experiments establishing methylation-to-phenotype causality, and brain tissue validation are essential prerequisites before any hypothesis can be formulated regarding relationships between genetic burden, epigenetic regulation, and disease manifestation. Only after such validation can integrated multi-omics approaches be considered for precision medicine applications in familial Alzheimer’s disease risk assessment.

## Figures and Tables

**Table 1:** Alzheimer’s disease candidate genes and their functional roles.

Gene	Chromosome Location (GRCh37/hg19)	Function in AD Pathology
*APOE*	chr19: 45,409,011–45,412,650	Amyloid-β metabolism and clearance
*PICALM*	chr11: 85,673,738–85,782,667	Endocytosis and tau pathology
*CLU*	chr8: 27,453,434–27,469,869	Amyloid clearance and chaperone activity
*CR1*	chr1: 207,494,290–207,667,439	Immune response and amyloid clearance
*BIN1*	chr2: 127,803,005–127,897,976	Tau pathology and endocytic trafficking
*ABCA7*	chr19: 1,040,673–1,084,340	Lipid metabolism and amyloid processing

## Data Availability

The raw genotype and DNA methylation data generated in this study are not publicly available due to privacy and ethical restrictions related to identifiable family genetic information but are available from the corresponding author upon reasonable request and with appropriate ethical approval. Access requests should include a detailed research proposal and evidence of relevant Institutional Review Board approval. Summary-level data supporting the findings of this study are available within the article and its Supplementary Materials (Supplementary Figures S1 and S2, Supplementary Tables S1 and S2).

## Supplementary Materials

The following supporting information can be downloaded at: https://www.mdpi.com/article/10.3390/jpm16020066/s1, Figure S1: Gene-Gene Correlation Matrix (Core Family, *n* = 7). Heatmap showing pairwise Pearson correlation coefficients between risk allele counts across six AD-associated genes (*APOE*, *PICALM*, *CLU*, *CR1*, *BIN1*, *ABCA7*). Color intensity represents correlation strength (red = positive, blue = negative, scale: −1.0 to +1.0). Diagonal shows perfect self-correlation (r = 1.00). Note: With only seven observations (*n* = 7), correlation coefficients are highly unstable—confidence intervals span ±0.4 to ±0.6, and a single data point can substantially alter results. For example, *APOE-CR1* shows r = 0.78, but this value could range from 0.2 to 0.95 in the true population. These values should be considered illustrative only and require validation in larger cohorts (*n* > 50) before drawing biological inferences about gene-gene interactions. Figure S2: Generational Analysis of Genetic Burden Scores (Core Family, *n* = 7). (a) Polygenic risk score trajectories across three generations. Red solid line represents mean GBS for all members, red dashed line shows *APOE* ε4 carriers (increasing from Gen I to Gen III), blue dashed line shows non-carriers (decreasing trend). Black star indicates the affected individual J-003 (GBS = 39, Generation I). Sample sizes per generation are indicated in boxes (*n* = 3 for Gen I, n=2 for Gen II and III). The V-shaped pattern shows Gen II with lowest burden (mean 43.0), followed by rebound in Gen III (mean 49.0). (b) Stacked bar chart showing gene-specific contributions to average GBS by generation. Numbers in colored segments indicate mean risk allele counts per gene. Total GBS per generation shown in yellow boxes above bars (48, 43, 49). *CR1* (orange) dominates across all generations (16.7–19.5 alleles), while *CLU* (teal) shows dramatic expansion in Gen III (8.0 alleles vs. 4.0–4.3 in earlier generations). *APOE* (red) remains stable at 2.0–3.5 alleles across generations. Table S1: Comparison of Genetic Burden Score (GBS) vs. GWAS-Based Polygenic Risk Score (PRS). This table provides a detailed methodological comparison between our candidate-gene GBS approach and traditional genome-wide PRS. Key distinctions include: (1) Variant coverage: GBS uses 320 SNPs across 6 genes vs. PRS using 50,000+ genome-wide variants; (2) Weighting: GBS employs unweighted allele counting (0/1/2) vs. PRS using effect-size weighted scores (β or OR); (3) Intended use: GBS for within-family relative comparison vs. PRS for population-level absolute risk prediction; (4) Output interpretation: GBS provides relative burden rankings within this family vs. PRS providing disease probabilities or percentile ranks; (5) Korean Chip coverage: 99.4% (318/320 variants) directly genotyped, with rs429358 and rs7412 determined independently. Table S2: Complete List of 320 SNPs Used for GBS Calculation with Individual Genotypes for All Seven Core Family Members. This comprehensive table lists all variants included in the genetic burden score analysis, providing: (1) SNP identifiers (rsID or chr:pos:ref:alt format); (2) Chromosomal positions (GRCh37/hg19 assembly); (3) Gene annotation (*APOE*: 9 SNPs including rs429358/rs7412; *PICALM*: 89 SNPs; *CLU*: 29 SNPs; *CR1*: 84 SNPs; *BIN1*: 8 SNPs; *ABCA7*: 101 SNPs); (4) Risk allele designation based on published GWAS (5) Data source (lead GWAS SNPs or LD proxy variants with r^2^ > 0.6 in East Asian populations); (6) Individual genotypes for all seven family members (J-003, J-005, J-006, J-011, J-025, J-012, J-017) in standard GT format (e.g., A/A, C/T, ./. for missing). Missing genotypes (./.) are indicated for a small number of variants that failed quality control in specific individuals.

## Abbreviations

AD	Alzheimer’s disease
GBS	Polygenic risk score
*PICALM*	Phosphatidylinositol-binding clathrin assembly protein
*CLU*	clusterin
*CR1*	Complement receptor 1
*BIN1*	Bridging integrator 1
*ABCA7*	ATP-binding cassette subfamily A member 7
SNP	Single nucleotide polymorphism
*APOE*	Apolipoprotein E
