# Supplementary figures and images for: Genetic Burden and APOE Methylation in a Korean Multi-Generational Alzheimer’s Disease Family: An Exploratory Multi-Omics Case Study
Source: J Pers Med. 2026 Jan 29;16(2):66. doi: 10.3390/jpm16020066 (PMC12941720; doi:10.3390/jpm16020066)

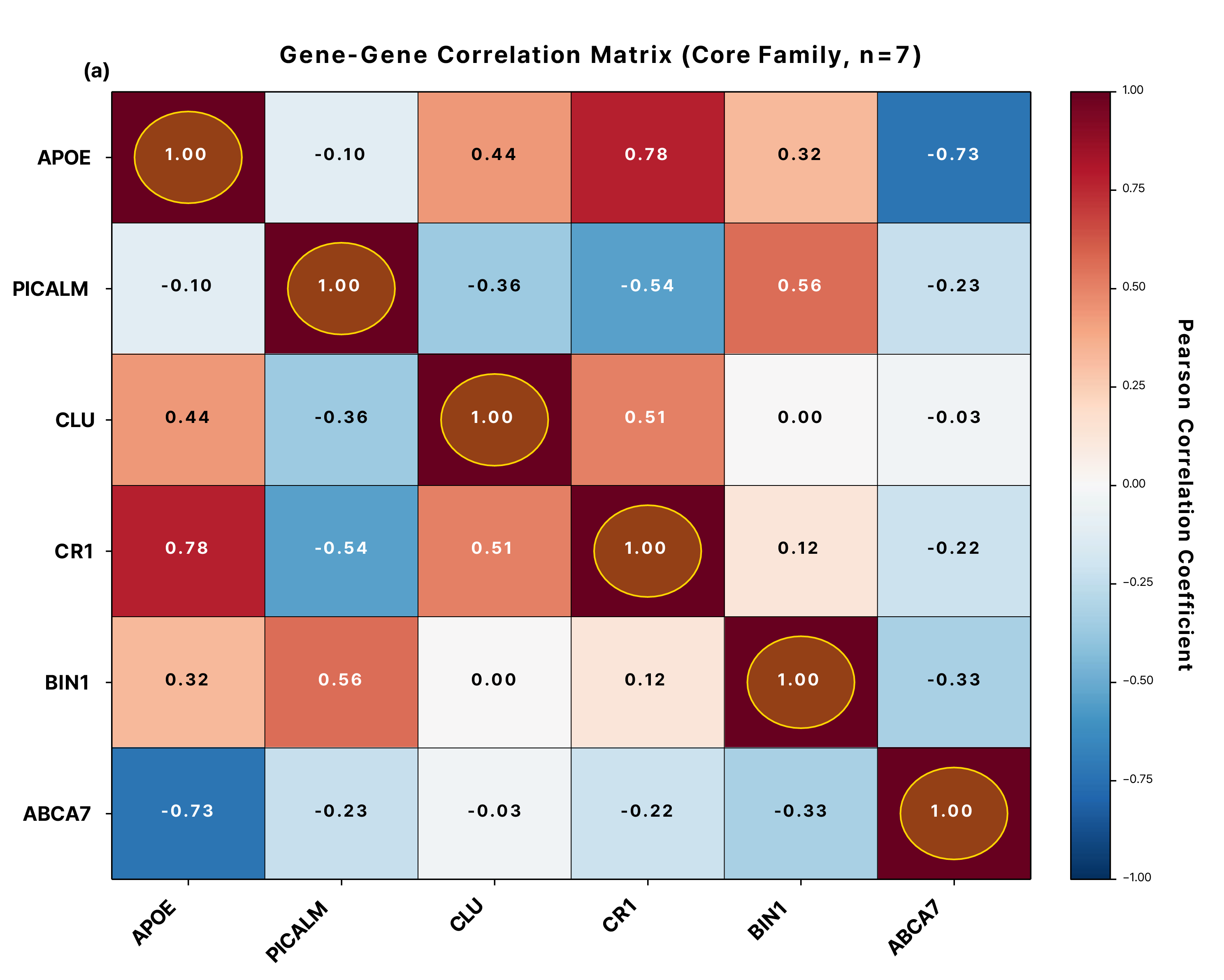

Supplement: Supplementary file 1 [file jpm-16-00066-s001.zip › Supplementary Figure S1.tif]

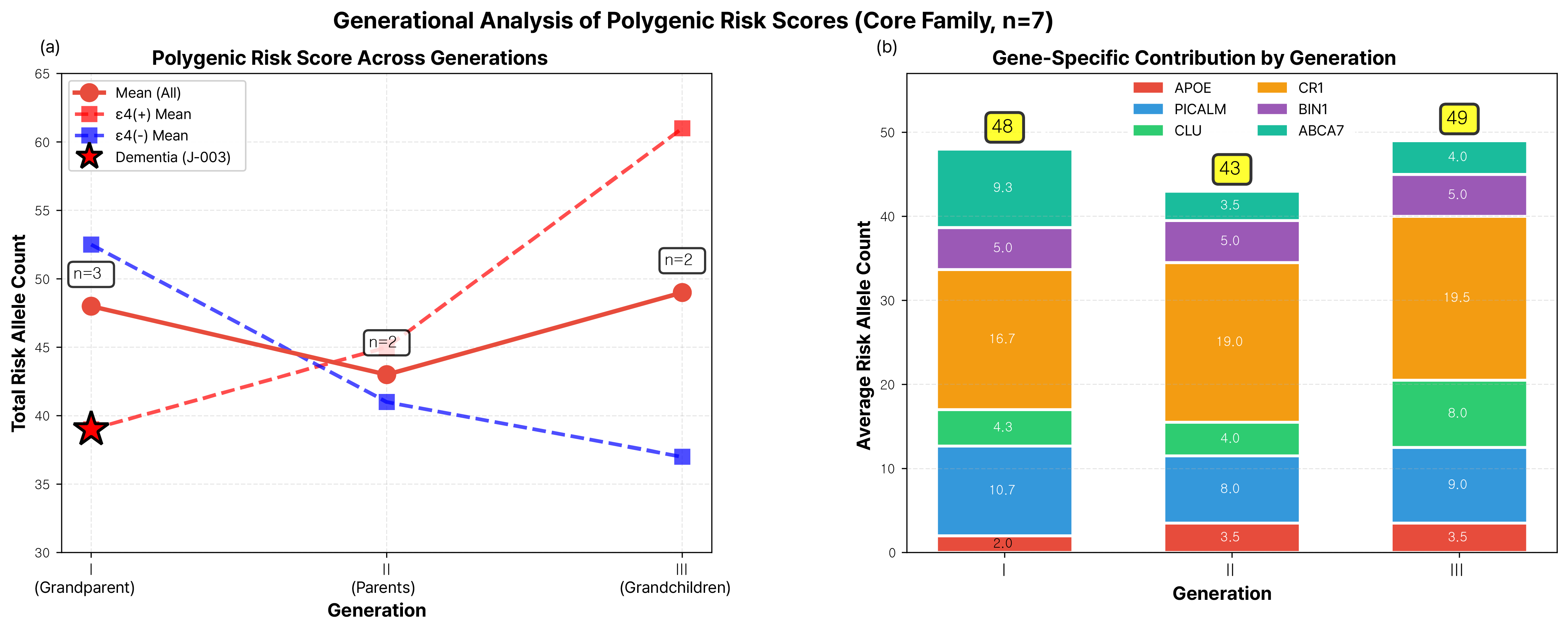

Supplement: Supplementary file 1 [file jpm-16-00066-s001.zip › Supplementary Figure S2.tif]
